# Crystal structure of the *meso* compound (2*R*,6*S*)-4-(5-bromo­pyrimidin-2-yl)-2,6-di­methyl­morpholine

**DOI:** 10.1107/S2056989026006158

**Published:** 2026-06-23

**Authors:** Paul R. Palme, Richard Goddard, Markus Leutzsch, Adrian Richter, Peter Imming, Rüdiger W. Seidel

**Affiliations:** a Institut für Pharmazie, Martin-Luther-Universität Halle-Wittenberg, Wolfgang-Langenbeck-Str. 4, 06120 Halle (Saale), Germany; b Max-Planck-Institut für Kohlenforschung, Kaiser-Wilhelm-Platz 1, 45470 Mülheim an der Ruhr, Germany

**Keywords:** crystal structure, Hirshfeld atom refinement, non-spherical atomic form factors, 2,6-di­methyl­morpholine, *meso* compound

## Abstract

The title compound, C_10_H_14_N_3_OBr, was prepared by a nucleophilic aromatic substitution reaction between 2,6-di­methyl­morpholine and 2-chloro­pyrimidine, followed by bromination of the pyrimidine ring. The compound crystallizes in the monoclinic system (space group *P*2_1_/c) with four mol­ecules in the unit cell (*Z* = 4). The mol­ecule exhibits approximate *C*_S_ point-group symmetry (r.m.s. deviation: 0.1072 Å). The arrangement of the mol­ecules in the solid state is dominated by close packing. C—H⋯N contacts between pyrimidinyl rings in adjacent mol­ecules with an *R*^2^_2_(6) motif are encountered, whereas the bromine atom does not exhibit any short contacts that could be regarded as halogen bonds.

## Abstract 2

The title compound, C_10_H_14_N_3_OBr, crystallizes in the monoclinic system (space group *P*2_1_/c, *Z* = 4) with the mol­ecule exhibiting approximate *C*_S_ point-group symmetry.

## 1. Chemical context

**Figure 1:**
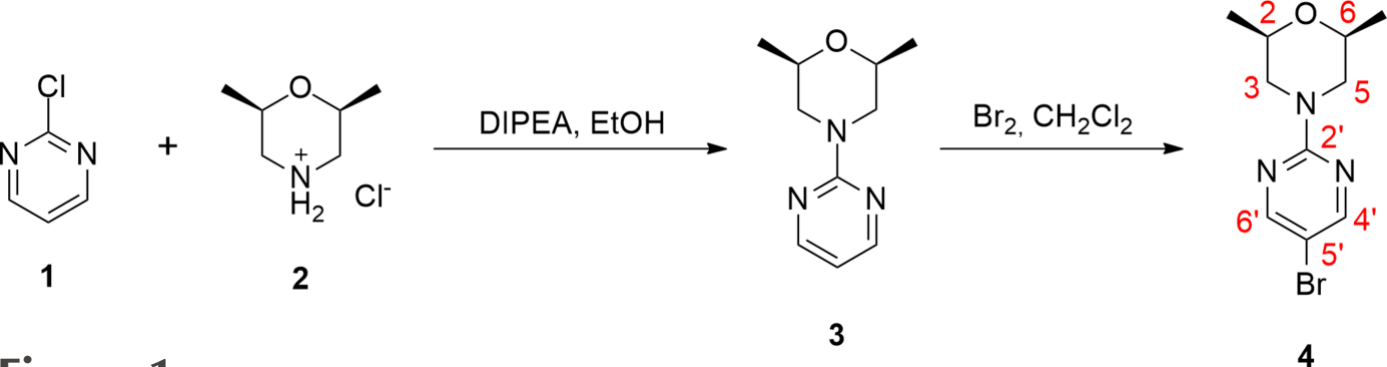
Two-step synthesis of **4**. DIPEA = *N*,*N*-Diiso­propyl­ethyl­amine (Hünig’s base).

2,6-Di­methyl­morpholine, usually the *cis* isomer, is a common building block in medicinal chemistry as it allows for modulating lipophilicity, basicity, metabolic stability and binding to the biological target. The anti­fungal agent amorolfin and the anti­neoplastic compound sonidegib are examples of approved and marketed drugs containing a *cis*-2,6-di­methyl­morpholine group. In the context of our anti­mycobacterial drug discovery efforts, 4-aryl­morpholine building blocks have attracted our inter­est (Palme *et al.*, 2025 ▸). We synthesized and crystallographically characterized (2*R*,6*S*)-4-(5-bromo­pyrimidin-2-yl)-2,6-di­methyl­morpholine (**4**) in two steps from commercially available starting materials (Fig. 1 ▸), adapting an established route (Cheprakova *et al.*, 2014 ▸). The first step was a nucleophilic aromatic substitution (S_N_Ar) reaction between 2-chloro­pyrimidine (**1**) and 2,6-di­methyl­morpholine hydro­chloride (**2**) in the presence of a base to yield 2,6-dimethyl-4-(pyrimidin-2-yl)morpholine (**3**; Hanyu *et al.*, 2009 ▸). [The configuration of 2,6-di­methyl­morpholine hydro­chloride as purchased was unspecified, but ^1^H and ^13^C NMR spectroscopy (see supporting information) indicate that only one isomer was present]. It is worth noting that Wei *et al.* (2019 ▸) reported the synthesis of **3** by a transition-metal-free cross-coupling reaction of 2-cyano­pyrimidine with 2,6-di­methyl­morpholine, and that, recently, Hall *et al.* (2025 ▸) described a one-pot sequential de­sulfonyl­ative fluorination of pyrimidine-2-sulfonyl fluoride followed by an S_N_Ar reaction with 2,6-di­methyl­morpholine. Finally, bromination of **3** in the second step gave compound **4** in good yield. Synthesis of **4** from 5-bromo-2-chloro­pyrimidine and 2,6-di­methyl­morpholine under S_N_Ar conditions has been described in the patent literature (Heng *et al.*, 2008 ▸; Yoshihara *et al.*, 2011 ▸; Wu *et al.*, 2015 ▸; You *et al.*, 2023 ▸; Shojaei *et al.*, 2023 ▸).
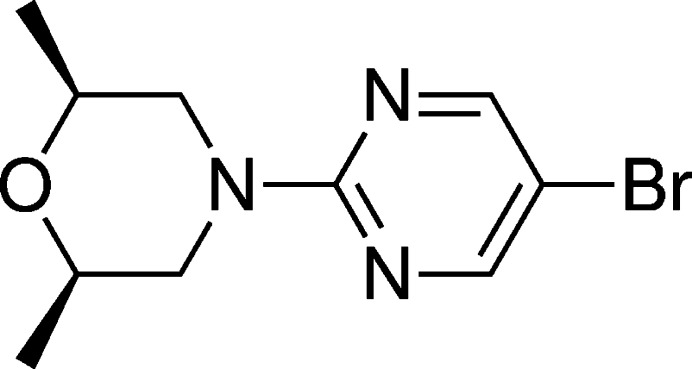


## 2. Structural commentary

**Figure 2:**
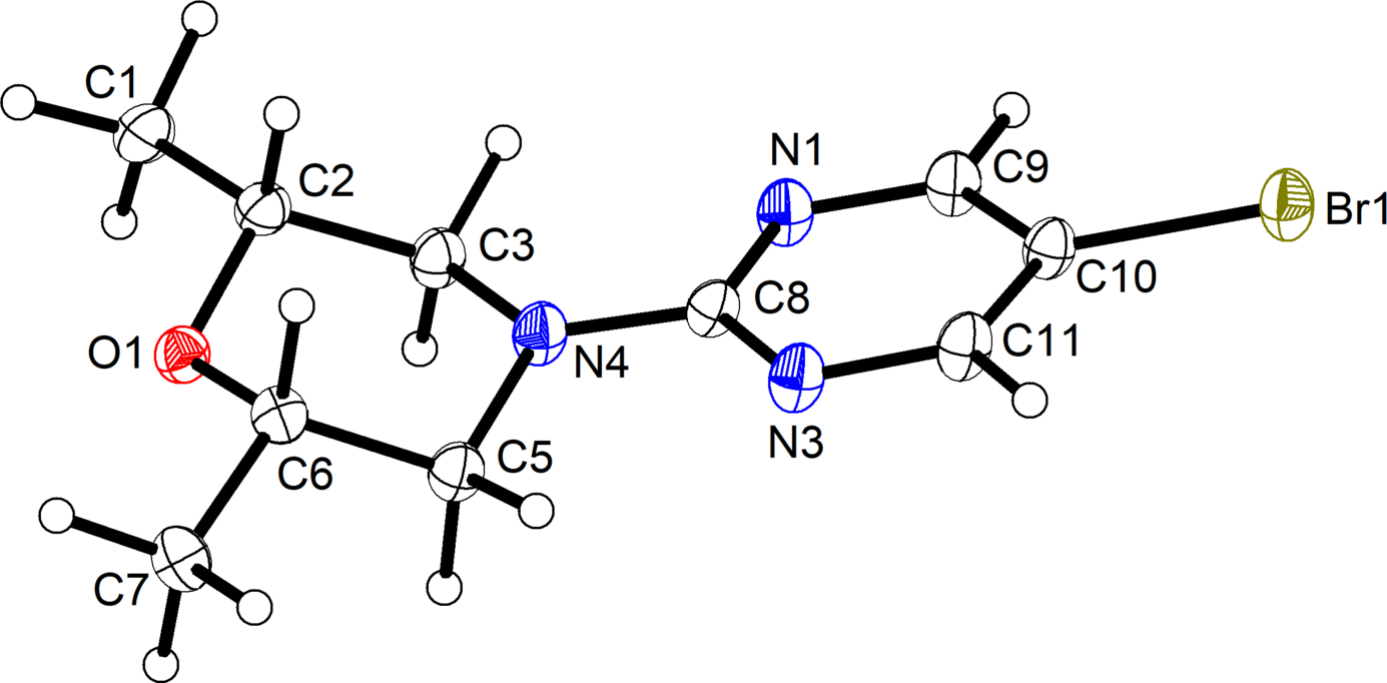
Mol­ecular structure of **4** in the crystal. Displacement ellipsoids are drawn at the 50% probability level. H atoms are represented as small spheres of arbitrary radius.

Fig. 2 ▸ shows the mol­ecular structure of **4** in the crystal. X-ray crystallography confirmed that the 2,6-di­methyl­morpholine ring is *cis*-configured. As expected, it adopts a chair conformation with the two methyl groups in equatorial positions. The 2,6-di­methyl­morpholine group and the pyrimidine ring are slightly inclined relative to one another about the C8—N4 bond, resulting in a r.m.s. deviation from exact mol­ecular *C*_S_ point group symmetry of 0.1072 Å. The geometry at N4 of the morpholine ring deviates marginally from planarity, as indicated by Σ(C—N—C) = 357.89 (9)°, which is barely smaller than 360° expected for an ideal planar coordination. The pyramidal height, *i.e.* the perpendicular distance of N4 to the plane specified by C3, C5 and C8, is small [0.1198 (6) Å]. This indicates that the lone pair of N4 is conjugated with the aromatic system of the pyrimidine ring.

The high-frequency shift of the ^1^H NMR signal assigned to the equatorial H atoms at C3 and C5 (4.45–4.33 ppm) can most likely be attributed to an anisotropic shielding effect exerted by the lone pairs of N1 and N3. Such a high-frequency shift of the morpholine H-3_eq_/H-5_eq_ signal was not observed for 2,6-dimethyl-4-phenyl­morpholine (Yuan *et al.*, 2024 ▸) or other *cis*-2,6-di­methyl­morpholine derivatives (Brügel, 1969 ▸). The C8—N4 bond is shorter than the corresponding C—N bond in 4-phenylmorpholine (WOXMOP; Jiang *et al.*, 2023 ▸) by 0.048 (2) Å.

## 3. Supra­molecular features

**Figure 3:**
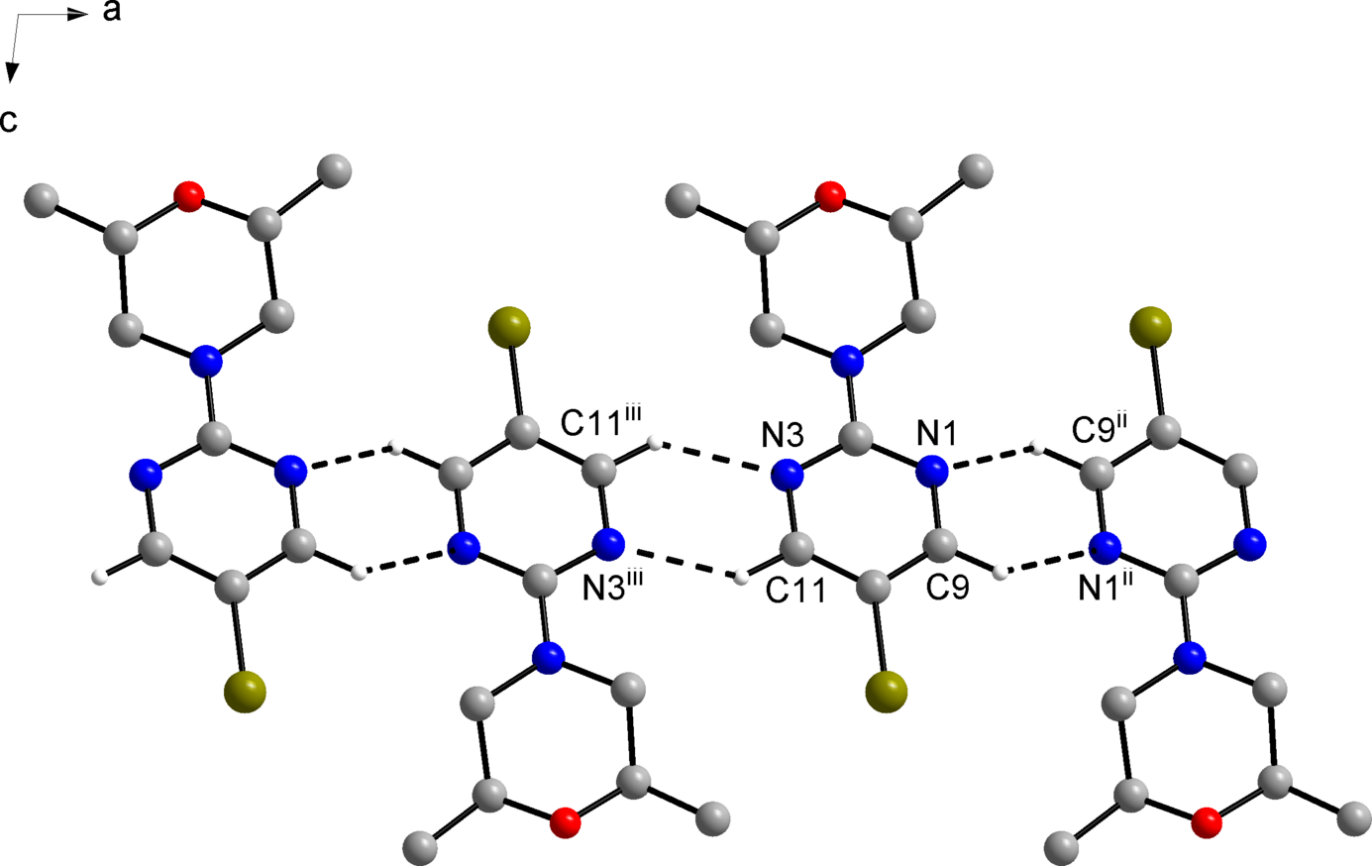
Part of the crystal structure of **4** viewed along the *b-*axis direction. H atoms not involved in C—H⋯N weak hydrogen bonds are omitted for clarity. Dashed lines represent weak hydrogen bonds. Colour scheme: C, grey; H, white; Br, dark yellow; N, blue; O, red. Symmetry codes: (ii) −*x* + 2, −*y* + 1, −*z* + 1; (iii) −*x* + 1, −*y* + 2, −*z* + 1.

The most prominent supra­mol­ecular feature in the crystal structure of **4** is weak C—H⋯N hydrogen bonding between the pyrimidine rings of adjacent symmetry-related mol­ecules (Fig. 3 ▸), resulting in centrosymmetric 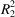
(6) motifs (Bernstein *et al.*, 1995 ▸). The geometric parameters listed in Table 1 ▸ suggest that the C11—H11⋯N3^iii^ hydrogen bond is more favourable than C9—H9⋯N1^ii^, as indicated by shorter H⋯*A* and *D*⋯*A* distances and a *D*—H⋯*A* angle closer to linearity. In addition, a C_meth­yl_—H⋯O_morpholine_ inter­mol­ecular short contact (C7—H7*A*⋯O1^i^) can be identified. Such contacts are ubiquitous in crystal structures of organic mol­ecules (Desiraju, 1995 ▸). Short contacts to bromine that could be inter­preted as halogen bonds are not encountered.

**Figure 4:**
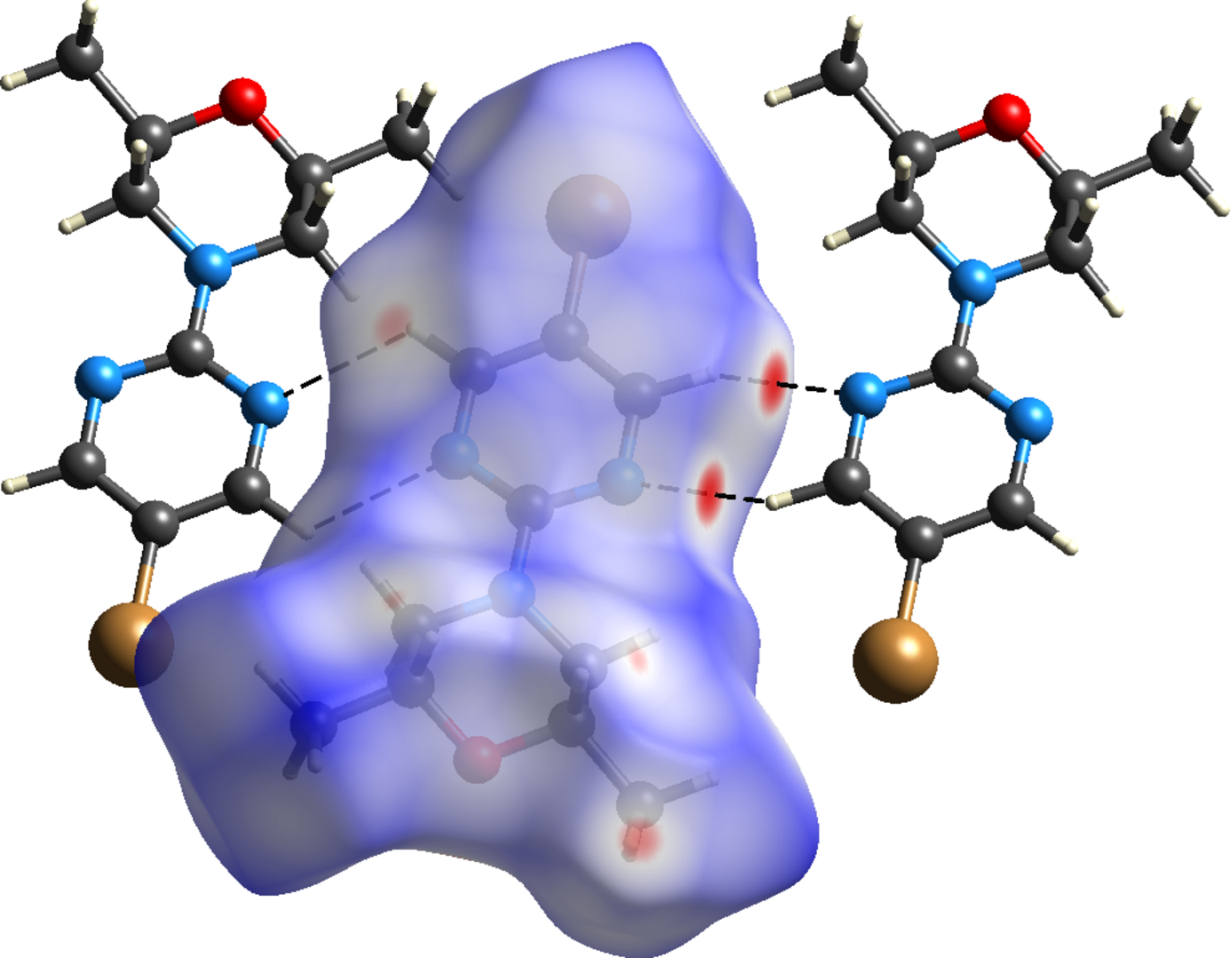
Hirshfeld surface mapped with *d*_norm_ for **4**. Colour scheme for the atoms: C, dark grey; H, white; Br, bronze; N, blue; O, red.

A Hirshfeld surface analysis was undertaken to investigate close inter­mol­ecular contacts and supra­mol­ecular assembly in the crystal structure in a more objective and quantitative manner (Spackman & Jayatilaka, 2009 ▸). Fig. 4 ▸ shows the Hirshfeld surface mapped with the normalized contact distance (*d*_norm_), whereby red, white and blue regions respectively indicate inter­mol­ecular contacts shorter, approximately equal and longer than the sum of the van der Waals radii (Bondi, 1964 ▸). Here, the two major red concave areas result from the C11—H11⋯N3^iii^ weak hydrogen bonds. Smaller red areas arise from the C9—H9⋯N1^ii^ and C7—H7*A*⋯O1^i^ short contacts as well as H⋯H inter­mol­ecular contacts involving the axial H atoms bonded to C2 and C6.

**Figure 5:**
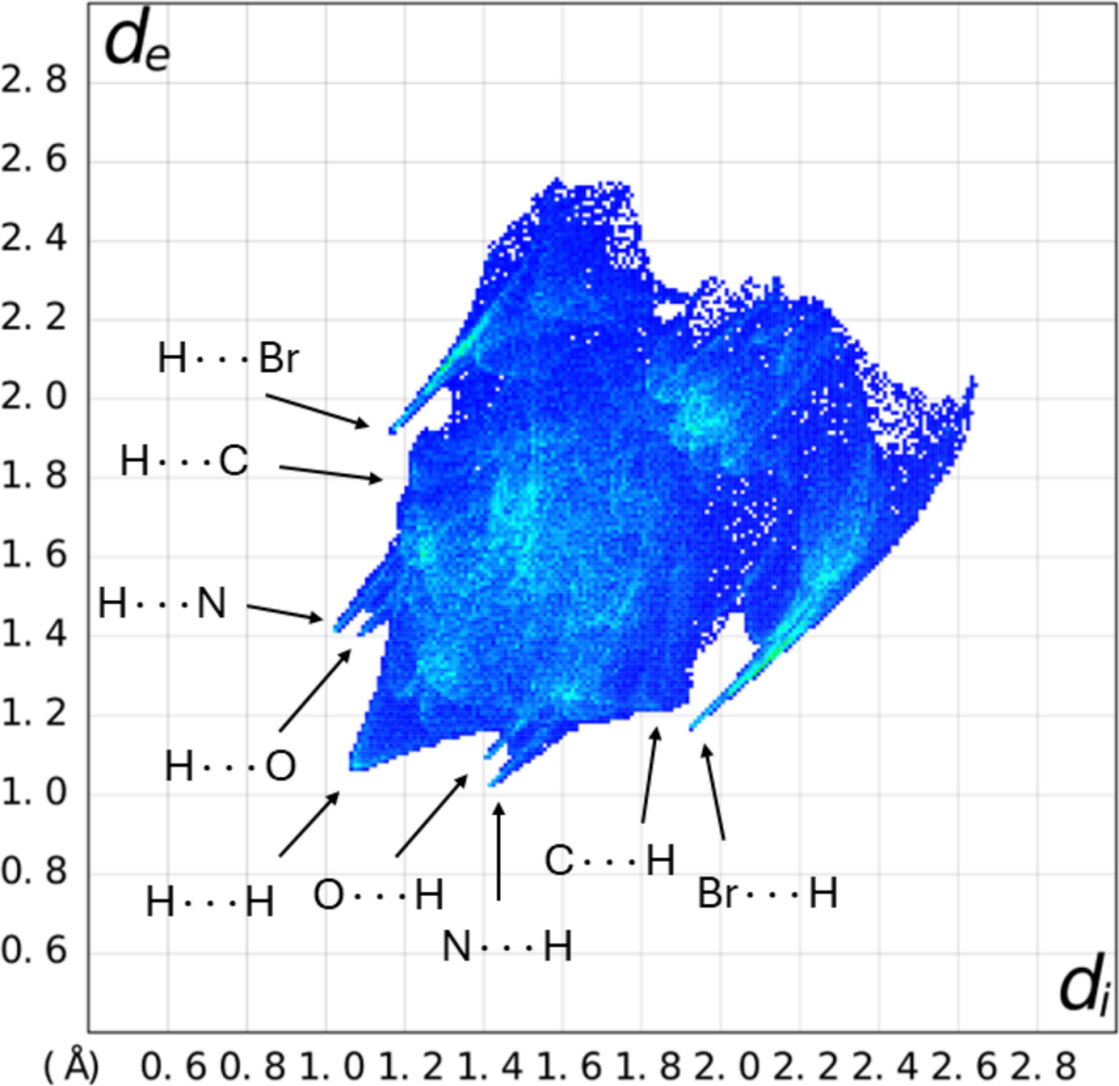
The two-dimensional fingerprint plot for **4**. *d*_i_ and *d*_e_ are the distances from the Hirshfeld surface to the nearest atoms inside and outside the surface, respectively. Dashed lines represent weak hydrogen bonds.

The corresponding two-dimensional fingerprint plot (Fig. 5 ▸) shows spikes from N⋯H/H⋯N (11.5% of the surface area included), O⋯H/H⋯O (5.2%) and Br⋯H/H⋯Br contacts (19.1%) as well as wings from C⋯H/H⋯C contacts (4.2%). A triangular feature on the diagonal characteristic of face-to-face aromatic stacking is not observed, and C⋯N/N⋯C and C⋯C contacts together only contribute 4.3% to the surface area included. H⋯H contacts account for 52.2% of the surface area. The tip on the diagonal centred at *d*_e_ + *d*_i_ < 2.4 Å (*i.e.* less than twice the van der Waals radius of hydrogen) mirrors the small and weak red spots at the morpholine H atoms in the axial 2,6-positions in the *d*_norm_ plot in Fig. 4 ▸. A slight asymmetry about the diagonal in the fingerprint plot is noticeable, in particular for the Br⋯H/H⋯Br spikes, which usually signals packing inefficiencies. Nonetheless, the packing index of 71% falls within the typical range observed for organic mol­ecular crystals (Kitajgorodskij, 1973 ▸).

## 4. Database survey

The crystal structure most closely related to that of **4** in the Cambridge Structural Database (CSD; Groom *et al.*, 2016 ▸) is the structure of 4-(5-bromo­pyrimidin-2-yl)morpholine (ROTXOP; Cheprakova *et al.*, 2014 ▸). The corresponding 5-nitro­pyrimidine derivative has also been crystallographically characterized (YILPEQ; Gorbunov *et al.*, 2013 ▸). Therein, the coordination at the morpholine N atom is virtually planar [Σ(C—N—C) = 360.0 (2)°], which can be attributed to the electron-withdrawing effect of the nitro group. As of June 2026, there are no examples of crystal structures containing 2,6-di­methyl­morpholine with N-bound unsubstituted aromatic groups in the CSD, but a relatively large number of crystal structures containing an unsubstituted 4-phenyl­morpholine moiety. In most of these crystal structures, the coordination at the morpholine N atom is markedly pyramidal, as in 4-phenyl­morpholine (WOXMOP; Jiang *et al.*, 2023 ▸).

## 5. Synthesis and crystallization

*General:* Starting materials were purchased and used as received. 2,6-Di­methyl­morpholine hydro­chloride was obtained from BLDpharm. Solvents were distilled before use. NMR spectra were recorded on an Agilent Technologies 600 MHz shielded VNMRS and an Agilent Technologies 400 MHz VNMRS spectrometer. Chemical shifts are reported relative to the residual solvent signal of chloro­form-*d* (δ_H_ = 7.26 ppm, δ_C_ = 77.16 ppm) or DMSO-*d*_6_ (δ_H_ = 2.50 ppm, δ_C_ = 39.51 ppm). Abbreviations: *s* = singlet, *d* = doublet, *t* = triplet, *dd* = doublet of doublets, *dqd* = doublet of quartet of doublets, *m* = multiplet. HRMS data were acquired on a Thermo Scientific Q Exactive GC Orbitrap GC-MS system.

*2,6-Dimethyl-4-(pyrimidin-2-yl)morpholine (**3**):* 2-Chloro­pyrimidine (**1**) (5.73 g, 50.0 mmol) and 2,6-di­methyl­morpholine hydro­chloride (**2**) (7.59 g, 50.0 mmol) were suspended in 50 mL of ethanol and 15 mL of DIPEA were added with stirring. The mixture was heated to reflux for 7 h. Subsequently, the solvent was removed under reduced pressure and the residue was taken up with ethyl acetate (50 mL). After washing successively with water (30 mL) and brine (30 mL), the organic layer was dried over magnesium sulfate and the solvent was evaporated under reduced pressure. The crude product was purified by flash chromatography (Inter­chim puriFlash^®^ 430) on silica gel using gradient elution with *n*-hepta­ne/ethyl acetate to yield **3** as a colourless oil (8.41 g, 44.0 mmol, 88%). ^1^H NMR (600 MHz, chloro­form-*d*) δ 8.31 (*d*, *J* = 4.8 Hz, 2H, H-4′/H-6′), 6.50 (*t*, *J* = 4.8 Hz, 1H, H-5′), 4.58–4.52 (*m*, 2H, H-3_eq_/H-5_eq_), 3.63 (*dqd*, *J* = 10.6, 6.3, 2.4 Hz, 2H, H-2_ax_/H-6_ax_), 2.60 (*dd*, *J* = 13.2, 10.6 Hz, 2H, H-3_ax_/H-5_ax_), 1.24 (*d*, *J* = 6.3 Hz, 6H, CH_3_) ppm. ^13^C{^1^H} NMR (151 MHz, chloro­form-*d*): δ 161.1 (C-2′), 157.8 (C-4′/C-6′), 110.1 (C-5′), 71.9 (C-2/C-6), 49.5 (C-3/C-5), 19.0 (CH_3_) ppm.

*(2R,6S)-4-(5-Bromo­pyrimidin-2-yl)-2,6-di­methyl­morpho­line (**4**):* Compound **3** (5.00 g, 26.1 mmol) was dissolved in 50 mL of di­chloro­methane and 0.80 mL (31.2 mmol) of bromine were added dropwise with stirring. After stirring for 12 h at room temperature, the progress of the reaction was checked by TLC. An additional 0.20 mL (7.8 mmol) of bromine was added and stirring was continued for 1 h. Subsequently, approx. 30 mL of a saturated aqueous sodium thio­sulfate solution were added, whereupon the mixture became colourless. The organic layer was separated and the aqueous phase was extracted with di­chloro­methane (2 × 30 mL) followed by ethyl acetate (1 × 30 mL). After drying over magnesium sulfate, the combined organic layers were evaporated to dryness to obtain compound **4** as a white solid (6.58 g, 24.2 mmol, 93%). ^1^H NMR (402 MHz, DMSO-*d*_6_) δ 8.45 (*s*, 2H, H-4′/H-6′), 4.45–4.33 (*m*, 2H, H-3_eq_/H-5_eq_), 3.53 (*dqd*, *J* = 10.7, 6.2, 2.5 Hz, 2H, H-2_ax_/H-6_ax_), 2.52 (*dd*, *J* = 13.2, 10.7 Hz, 2H, H-3_ax_/H-5_ax_), 1.13 (*d*, *J* = 6.2 Hz, 6H, CH_3_) ppm. ^13^C{^1^H} APT NMR (101 MHz, DMSO-*d*_6_): δ 159.2 (C-2′), 157.9 (C-4′/C-6′), 105.5 (C-5′), 70.8 (C-2/C-6), 48.9 (C-3/C-5), 18.6 (CH_3_) ppm. HRMS(EI): *m*/*z* calculated for C_10_H_14_N_3_OBr^+^ 271.031486 [*M*]^+^, found 271.031420. Crystals suitable for single-crystal X-ray diffraction analysis were grown from a solution in di­chloro­methane by slow evaporation of the solvent at ambient conditions.

## 6. Refinement details

Crystal data, data collection and structure refinement details are summarized in Table 2 ▸. The crystal structure was initially refined with *SHELXL* (Sheldrick, 2015*b* ▸). Subsequently, Hirshfeld atom refinement was performed with *NoSpherA2* (Kleemiss *et al.*, 2021 ▸) in *OLEX2* (Dolomanov *et al.*, 2009 ▸). *ORCA* 6.1 (Neese, 2025 ▸) was used to calculate the electron density at the B3LYP/def2-TZVPP level of theory (Becke, 1993 ▸; Lee *et al.*, 1988 ▸; Weigend & Ahlrichs, 2005 ▸), which was partitioned into Hirshfeld atoms and converted *via* Fourier transform into atomic form factors (Midgley *et al.*, 2021 ▸). Least-squares refinements against the non-spherical atomic form factors thus obtained were carried out with *olex2.refine* (Bourhis *et al.*, 2015 ▸). Anisotropic atomic displacement parameters (ADPs) were introduced for all non-H atoms. Positions and isotropic ADPs of H atoms were refined freely.

The deviation from mol­ecular point-group symmetry was calculated with *MOLSYM* in *PLATON* (Spek, 2009 ▸) using the atomic weighting mode. Hirshfeld surface analysis was conducted with *CrystalExplorer 21* (Spackman *et al.*, 2021 ▸), which by default applies neutron-derived values for *X*—H bond lengths (Allen & Bruno, 2010 ▸).

## Supplementary Material

Crystal structure: contains datablock(s) I, global. DOI: 10.1107/S2056989026006158/meu2003sup1.cif

Structure factors: contains datablock(s) I. DOI: 10.1107/S2056989026006158/meu2003Isup2.hkl

Supporting information file. DOI: 10.1107/S2056989026006158/meu2003Isup3.cdx

1H and 13C NMR spectra of compound 2. DOI: 10.1107/S2056989026006158/meu2003sup4.pdf

1H and 13C NMR spectra of compound 3. DOI: 10.1107/S2056989026006158/meu2003sup5.pdf

1H and 13C NMR and NOESY spectra of compound 4. DOI: 10.1107/S2056989026006158/meu2003sup6.pdf

CCDC reference: 2561790

Additional supporting information:  crystallographic information; 3D view; checkCIF report

## Figures and Tables

**Table 1:** Hydrogen-bond geometry (Å, °)

*D*—H⋯*A*	*D*—H	H⋯*A*	*D*⋯*A*	*D*—H⋯*A*
C7—H7*A*⋯O1^i^	1.078 (12)	2.499 (12)	3.4295 (9)	143.9 (8)
C9—H9⋯N1^ii^	1.065 (10)	2.580 (10)	3.2779 (9)	122.5 (7)
C11—H11⋯N3^iii^	1.095 (11)	2.423 (11)	3.3610 (9)	142.8 (8)

Symmetry codes: (i) 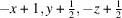; (ii) 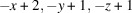; (iii) 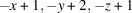.

**Table 2:** Experimental details

Crystal data	
Chemical formula	C_10_H_14_BrN_3_O
*M*_r_	272.15
Crystal system, space group	Monoclinic, *P*2_1_/*c*
Temperature (K)	100
*a*, *b*, *c* (Å)	10.3934 (6), 4.2323 (2), 25.8356 (14)
β (°)	97.076 (3)
*V* (Å^3^)	1127.8 (1)
*Z*	4
Radiation type	Mo *K*α
μ (mm^−1^)	3.63
Crystal size (mm)	0.18 × 0.10 × 0.07

Data collection	
Diffractometer	Bruker AXS D8 VENTURE
Absorption correction	Gaussian (*SADABS*; Krause *et al.*, 2015 ▸)
*T*_min_, *T*_max_	0.679, 0.860
No. of measured, independent and observed [*I* ≥ 2u(*I*)] reflections	97547, 3622, 3383
*R*_int_	0.042
(sin θ/λ)_max_ (Å^−1^)	0.727

Refinement	
*R*[*F*^2^ > 2σ(*F*^2^)], *wR*(*F*^2^), *S*	0.013, 0.030, 1.06
No. of reflections	3622
No. of parameters	192
H-atom treatment	All H-atom parameters refined
Δρ_max_, Δρ_min_ (e Å^−3^)	0.33, −0.24

Computer programs: *APEX6* (Bruker, 2024 ▸), *SAINT* (Bruker, 2019 ▸), *SHELXT* (Sheldrick, 2015*a* ▸), *OLEX2.refine* (Bourhis *et al.*, 2015 ▸), *DIAMOND* (Brandenburg, 2018 ▸) and *publCIF* (Westrip, 2010 ▸).

## supplementary crystallographic information

### (2R,6S)-4-(5-Bromopyrimidin-2-yl)-2,6-dimethylmorpholine Crystal data

**Table:**

C_10_H_14_BrN_3_O	*F*(000) = 551.505
*M**_r_* = 272.15	*D*_x_ = 1.603 Mg m^−^^3^
Monoclinic, *P*2_1_/*c*	Mo *K*α radiation, λ = 0.71073 Å
*a* = 10.3934 (6) Å	Cell parameters from 9300 reflections
*b* = 4.2323 (2) Å	θ = 3.2–31.0°
*c* = 25.8356 (14) Å	µ = 3.63 mm^−^^1^
β = 97.076 (3)°	*T* = 100 K
*V* = 1127.8 (1) Å^3^	Prism, colourless
*Z* = 4	0.18 × 0.10 × 0.07 mm

### (2R,6S)-4-(5-Bromopyrimidin-2-yl)-2,6-dimethylmorpholine Data collection

**Table:**

Bruker AXS D8 VENTURE diffractometer	3622 independent reflections
Radiation source: IµS Diamond	3383 reflections with *I*≥ 2u(*I*)
Incoatec Helios mirrors monochromator	*R*_int_ = 0.042
Detector resolution: 7.391 pixels mm^-1^	θ_max_ = 31.1°, θ_min_ = 2.0°
φ and ω scans	*h* = −15→15
Absorption correction: gaussian (SADABS; Krause *et al.*, 2015)	*k* = −6→6
*T*_min_ = 0.679, *T*_max_ = 0.860	*l* = −37→37
97547 measured reflections	

### (2R,6S)-4-(5-Bromopyrimidin-2-yl)-2,6-dimethylmorpholine Refinement

**Table:**

Refinement on *F*^2^	Primary atom site location: dual
Least-squares matrix: full	Secondary atom site location: difference Fourier map
*R*[*F*^2^ > 2σ(*F*^2^)] = 0.013	Hydrogen site location: difference Fourier map
*wR*(*F*^2^) = 0.030	All H-atom parameters refined
*S* = 1.06	*w* = 1/[σ^2^(*F*_o_^2^) + (0.0129*P*)^2^ + 0.1241*P*] where *P* = (*F*_o_^2^ + 2*F*_c_^2^)/3
3622 reflections	(Δ/σ)_max_ = −0.0001
192 parameters	Δρ_max_ = 0.33 e Å^−^^3^
0 restraints	Δρ_min_ = −0.24 e Å^−^^3^
0 constraints	

### (2R,6S)-4-(5-Bromopyrimidin-2-yl)-2,6-dimethylmorpholine Special details

**Table:**

Experimental. Crystal mounted on a MiTeGen loop using Perfluoropolyether PFO-XR75
Refinement. Refinement using NoSpherA2, an implementation of NOn-SPHERical Atom-form-factors in Olex2. Please cite: F. Kleemiss *et al.* Chem. Sci. DOI 10.1039/D0SC05526C - 2021 NoSpherA2 implementation of HAR makes use of tailor-made aspherical atomic form factors calculated on-the-fly from a Hirshfeld-partitioned electron density (ED) - not from spherical-atom form factors.The ED is calculated from a gaussian basis set single determinant SCF wavefunction - either Hartree-Fock or DFT using selected funtionals - for a fragment of the crystal. This fragment can be embedded in an electrostatic crystal field by employing cluster charges or modelled using implicit solvation models, depending on the software used.The following options were used: SOFTWARE: ORCA 6.1 PARTITIONING: NoSpherA2 INT ACCURACY: Normal METHOD: B3LYP BASIS SET: def2-TZVPP CHARGE: 0 MULTIPLICITY: 1 DATE: 2026-04-01_20-42-29The minimum and maximum estimated transmissions from the multi-scan scaling are 0.6613 and 0.8743 (SADABS).

### (2R,6S)-4-(5-Bromopyrimidin-2-yl)-2,6-dimethylmorpholine Fractional atomic coordinates and isotropic or equivalent isotropic displacement parameters (Å^2^)

**Table:**

	*x*	*y*	*z*	*U*_iso_*/*U*_eq_	
C1	0.86770 (7)	0.35247 (18)	0.28616 (3)	0.02637 (14)	
H1A	0.8346 (11)	0.407 (2)	0.2447 (5)	0.049 (3)*	
H1B	0.8798 (11)	0.100 (2)	0.2894 (4)	0.048 (3)*	
H1C	0.9610 (11)	0.460 (3)	0.2975 (4)	0.051 (3)*	
C2	0.76932 (6)	0.47733 (16)	0.31971 (3)	0.02041 (12)	
H2	0.7597 (9)	0.736 (2)	0.3144 (4)	0.034 (2)*	
C3	0.80623 (6)	0.40775 (17)	0.37755 (3)	0.02130 (12)	
H3A	0.8193 (10)	0.150 (2)	0.3831 (4)	0.040 (3)*	
H3B	0.8993 (9)	0.525 (2)	0.3919 (4)	0.034 (2)*	
C5	0.57364 (6)	0.42407 (17)	0.38639 (3)	0.02103 (12)	
H5A	0.5027 (10)	0.546 (2)	0.4076 (4)	0.039 (2)*	
H5B	0.5608 (10)	0.170 (2)	0.3940 (4)	0.039 (3)*	
C6	0.54734 (6)	0.48648 (16)	0.32811 (3)	0.01974 (12)	
H6	0.5531 (8)	0.746 (2)	0.3220 (3)	0.032 (2)*	
C7	0.41680 (7)	0.36042 (17)	0.30472 (3)	0.02331 (13)	
H7A	0.3993 (11)	0.410 (3)	0.2635 (5)	0.056 (3)*	
H7B	0.3402 (11)	0.467 (3)	0.3244 (5)	0.053 (3)*	
H7C	0.4119 (11)	0.106 (2)	0.3097 (4)	0.048 (3)*	
C8	0.73225 (6)	0.65197 (15)	0.45507 (2)	0.01870 (11)	
C9	0.88456 (6)	0.79021 (17)	0.52262 (3)	0.02176 (12)	
H9	0.9834 (10)	0.791 (3)	0.5395 (4)	0.043 (2)*	
C10	0.78849 (6)	0.91794 (15)	0.54884 (2)	0.01989 (12)	
C11	0.66181 (6)	0.90051 (17)	0.52476 (3)	0.02281 (13)	
H11	0.5802 (10)	0.999 (3)	0.5425 (4)	0.047 (3)*	
Br1	0.829245 (7)	1.105440 (18)	0.615057 (3)	0.02623 (3)	
N1	0.85771 (5)	0.65779 (14)	0.47578 (2)	0.02223 (11)	
N3	0.63261 (5)	0.76677 (15)	0.47811 (2)	0.02287 (11)	
N4	0.70440 (5)	0.52684 (15)	0.40639 (2)	0.02233 (11)	
O1	0.64614 (4)	0.34162 (11)	0.302198 (18)	0.02004 (9)	

### (2R,6S)-4-(5-Bromopyrimidin-2-yl)-2,6-dimethylmorpholine Atomic displacement parameters (Å^2^)

**Table:**

	*U*^11^	*U*^22^	*U*^33^	*U*^12^	*U*^13^	*U*^23^
C1	0.0264 (3)	0.0294 (4)	0.0253 (3)	−0.0036 (3)	0.0111 (3)	−0.0039 (3)
C2	0.0226 (3)	0.0190 (3)	0.0205 (3)	−0.0011 (2)	0.0062 (2)	−0.0007 (2)
C3	0.0178 (3)	0.0270 (3)	0.0197 (3)	0.0014 (2)	0.0045 (2)	−0.0018 (2)
C5	0.0168 (3)	0.0271 (3)	0.0194 (3)	0.0011 (2)	0.0027 (2)	0.0002 (2)
C6	0.0206 (3)	0.0179 (3)	0.0203 (3)	0.0019 (2)	0.0011 (2)	0.0004 (2)
C7	0.0212 (3)	0.0234 (3)	0.0244 (3)	0.0028 (2)	−0.0009 (2)	−0.0021 (2)
C8	0.0149 (3)	0.0239 (3)	0.0175 (3)	0.0030 (2)	0.0029 (2)	0.0010 (2)
C9	0.0153 (3)	0.0303 (3)	0.0196 (3)	0.0036 (2)	0.0019 (2)	−0.0023 (2)
C10	0.0182 (3)	0.0245 (3)	0.0173 (3)	0.0035 (2)	0.0033 (2)	−0.0004 (2)
C11	0.0169 (3)	0.0325 (3)	0.0193 (3)	0.0064 (2)	0.0036 (2)	−0.0016 (3)
Br1	0.02647 (4)	0.03261 (4)	0.01976 (4)	0.00209 (3)	0.00346 (2)	−0.00558 (3)
N1	0.0148 (2)	0.0317 (3)	0.0204 (3)	0.0039 (2)	0.00277 (19)	−0.0036 (2)
N3	0.0151 (2)	0.0347 (3)	0.0190 (2)	0.0052 (2)	0.00271 (19)	−0.0018 (2)
N4	0.0155 (2)	0.0329 (3)	0.0189 (3)	0.0012 (2)	0.00342 (19)	−0.0032 (2)
O1	0.0220 (2)	0.0190 (2)	0.0194 (2)	0.00032 (17)	0.00371 (17)	−0.00088 (17)

### (2R,6S)-4-(5-Bromopyrimidin-2-yl)-2,6-dimethylmorpholine Geometric parameters (Å, º)

**Table:**

C1—H1A	1.108 (11)	C6—C7	1.5128 (9)
C1—H1B	1.079 (10)	C6—O1	1.4310 (8)
C1—H1C	1.078 (11)	C7—H7A	1.078 (12)
C1—C2	1.5147 (9)	C7—H7B	1.094 (11)
C2—H2	1.105 (10)	C7—H7C	1.085 (10)
C2—C3	1.5250 (10)	C8—N1	1.3473 (8)
C2—O1	1.4252 (8)	C8—N3	1.3471 (8)
C3—H3A	1.107 (10)	C8—N4	1.3626 (8)
C3—H3B	1.109 (10)	C9—H9	1.065 (10)
C3—N4	1.4572 (8)	C9—C10	1.3838 (9)
C5—H5A	1.101 (10)	C9—N1	1.3315 (9)
C5—H5B	1.104 (10)	C10—C11	1.3872 (9)
C5—C6	1.5201 (9)	C10—Br1	1.8860 (7)
C5—N4	1.4595 (8)	C11—H11	1.095 (11)
C6—H6	1.111 (10)	C11—N3	1.3321 (9)
			
H1B—C1—H1A	107.5 (8)	O1—C6—C5	109.70 (5)
H1C—C1—H1A	109.7 (8)	O1—C6—H6	107.6 (4)
H1C—C1—H1B	107.6 (8)	O1—C6—C7	108.79 (5)
C2—C1—H1A	109.2 (6)	H7A—C7—C6	111.1 (6)
C2—C1—H1B	112.4 (6)	H7B—C7—C6	109.7 (6)
C2—C1—H1C	110.3 (6)	H7B—C7—H7A	109.6 (9)
H2—C2—C1	109.3 (5)	H7C—C7—C6	110.8 (6)
C3—C2—C1	112.79 (6)	H7C—C7—H7A	107.7 (8)
C3—C2—H2	108.7 (5)	H7C—C7—H7B	107.8 (8)
O1—C2—C1	108.73 (5)	N3—C8—N1	125.24 (6)
O1—C2—H2	107.2 (5)	N4—C8—N1	117.32 (5)
O1—C2—C3	109.93 (5)	N4—C8—N3	117.42 (6)
H3A—C3—C2	109.2 (6)	C10—C9—H9	121.0 (5)
H3B—C3—C2	110.2 (5)	N1—C9—H9	117.2 (5)
H3B—C3—H3A	108.0 (7)	N1—C9—C10	121.75 (6)
N4—C3—C2	108.86 (5)	C11—C10—C9	117.51 (6)
N4—C3—H3A	111.1 (5)	Br1—C10—C9	120.90 (5)
N4—C3—H3B	109.5 (5)	Br1—C10—C11	121.60 (5)
H5B—C5—H5A	105.4 (7)	H11—C11—C10	122.2 (6)
C6—C5—H5A	111.2 (5)	N3—C11—C10	121.75 (6)
C6—C5—H5B	109.6 (5)	N3—C11—H11	116.0 (6)
N4—C5—H5A	109.6 (5)	C9—N1—C8	116.93 (6)
N4—C5—H5B	110.8 (5)	C11—N3—C8	116.82 (6)
N4—C5—C6	110.17 (5)	C5—N4—C3	114.79 (5)
H6—C6—C5	107.9 (4)	C8—N4—C3	121.42 (5)
C7—C6—C5	112.17 (6)	C8—N4—C5	121.68 (5)
C7—C6—H6	110.6 (4)	C6—O1—C2	110.32 (5)
			
C1—C2—C3—N4	−178.16 (6)	C7—C6—C5—N4	175.48 (6)
C1—C2—O1—C6	−172.02 (5)	C8—N1—C9—C10	−0.37 (7)
C2—C3—N4—C5	51.43 (6)	C8—N3—C11—C10	−0.71 (7)
C2—C3—N4—C8	−144.83 (5)	C9—C10—C11—N3	−0.09 (8)
C2—O1—C6—C5	−62.59 (5)	C9—N1—C8—N3	−0.53 (8)
C2—O1—C6—C7	174.37 (5)	C9—N1—C8—N4	177.77 (6)
C3—C2—O1—C6	64.06 (6)	C11—C10—C9—N1	0.66 (8)
C3—N4—C5—C6	−50.89 (6)	C11—N3—C8—N1	1.07 (8)
C3—N4—C8—N1	−0.77 (7)	C11—N3—C8—N4	−177.23 (6)
C3—N4—C8—N3	177.67 (6)	Br1—C10—C9—N1	−179.78 (5)
C5—N4—C8—N1	161.85 (6)	Br1—C10—C11—N3	−179.64 (5)
C5—N4—C8—N3	−19.71 (7)	N4—C3—C2—O1	−56.64 (6)
C6—C5—N4—C8	145.42 (5)	N4—C5—C6—O1	54.47 (6)

### (2R,6S)-4-(5-Bromopyrimidin-2-yl)-2,6-dimethylmorpholine Hydrogen-bond geometry (Å, º)

**Table:**

*D*—H···*A*	*D*—H	H···*A*	*D*···*A*	*D*—H···*A*
C7—H7*A*···O1^i^	1.078 (12)	2.499 (12)	3.4295 (9)	143.9 (8)
C9—H9···N1^ii^	1.065 (10)	2.580 (10)	3.2779 (9)	122.5 (7)
C11—H11···N3^iii^	1.095 (11)	2.423 (11)	3.3610 (9)	142.8 (8)

Symmetry codes: (i) −*x*+1, *y*+1/2, −*z*+1/2; (ii) −*x*+2, −*y*+1, −*z*+1; (iii) −*x*+1, −*y*+2, −*z*+1.
