# Supplementary figures and images for: Crystal structure of the meso compound (2R,6S)-4-(5-bromo­pyrimidin-2-yl)-2,6-di­methyl­morpholine
Source: Acta Crystallogr E Crystallogr Commun. 2026 Jun 23;82(Pt 7):768–72. doi: 10.1107/S2056989026006158 (PMC13330847; doi:10.1107/S2056989026006158)

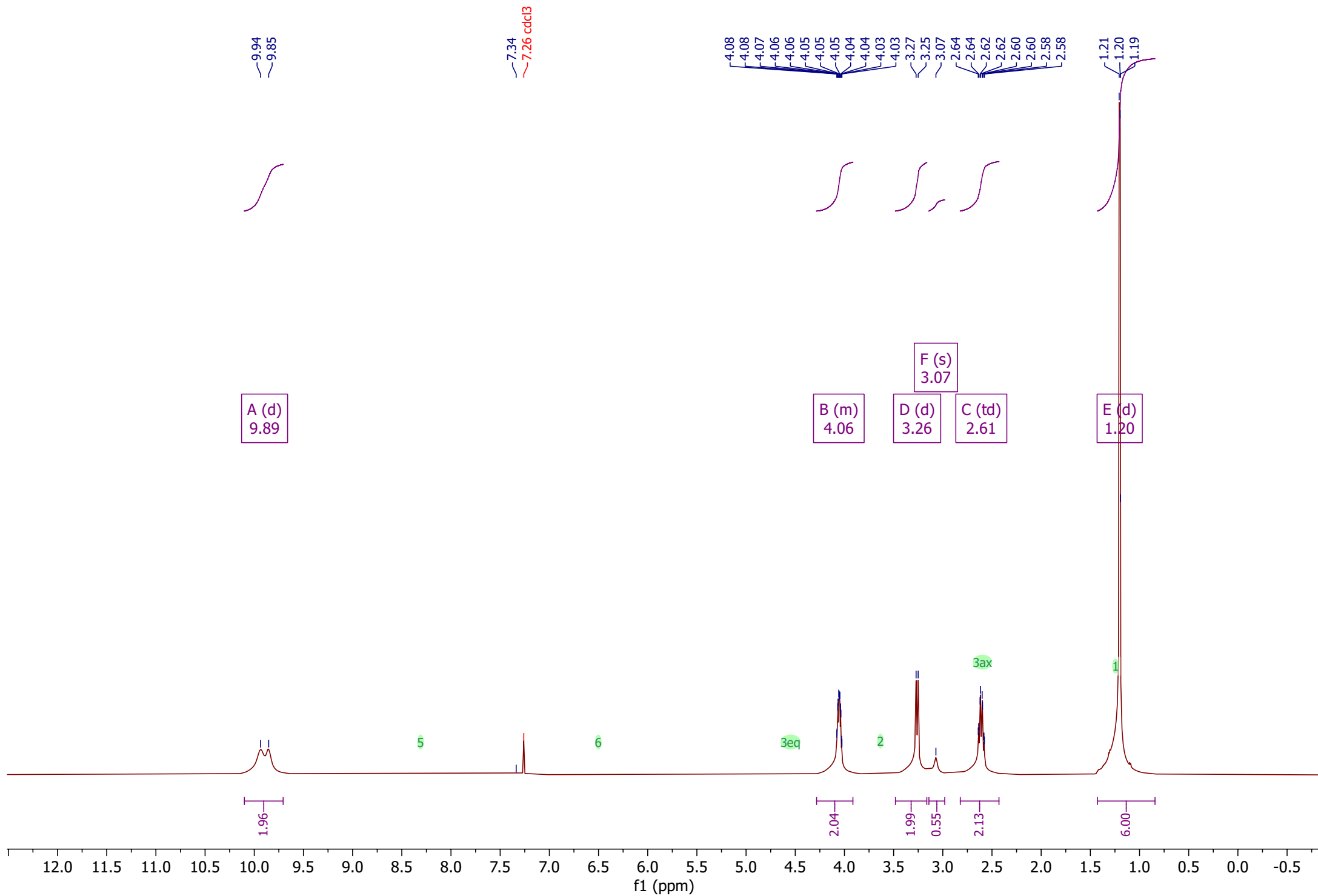

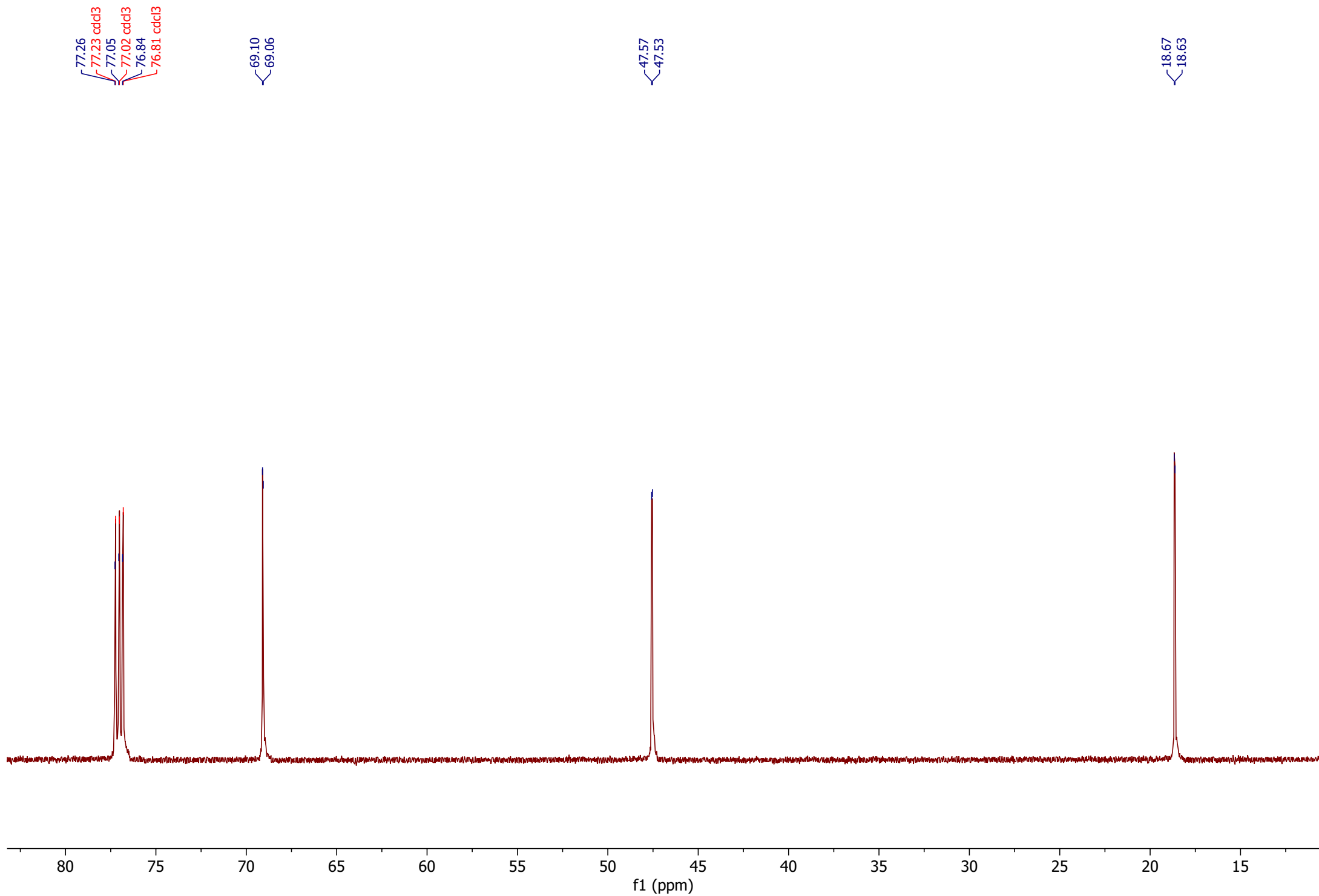

Supplement: Supplementary file 4 [file e-82-00768-sup4.pdf]

$^1\text{H}$  NMR (600 MHz,  $\text{CDCl}_3$ )  $\delta$  8.31 (d,  $J = 4.8$  Hz, 2H), 6.50 (t,  $J = 4.8$  Hz, 1H), 4.58 – 4.52 (m, 2H), 3.63 (dq,  $J = 10.6, 6.3, 2.4$  Hz, 2H), 2.60 (dd,  $J = 13.2, 10.6$  Hz, 2H), 1.24 (d,  $J = 6.3$  Hz, 6H).

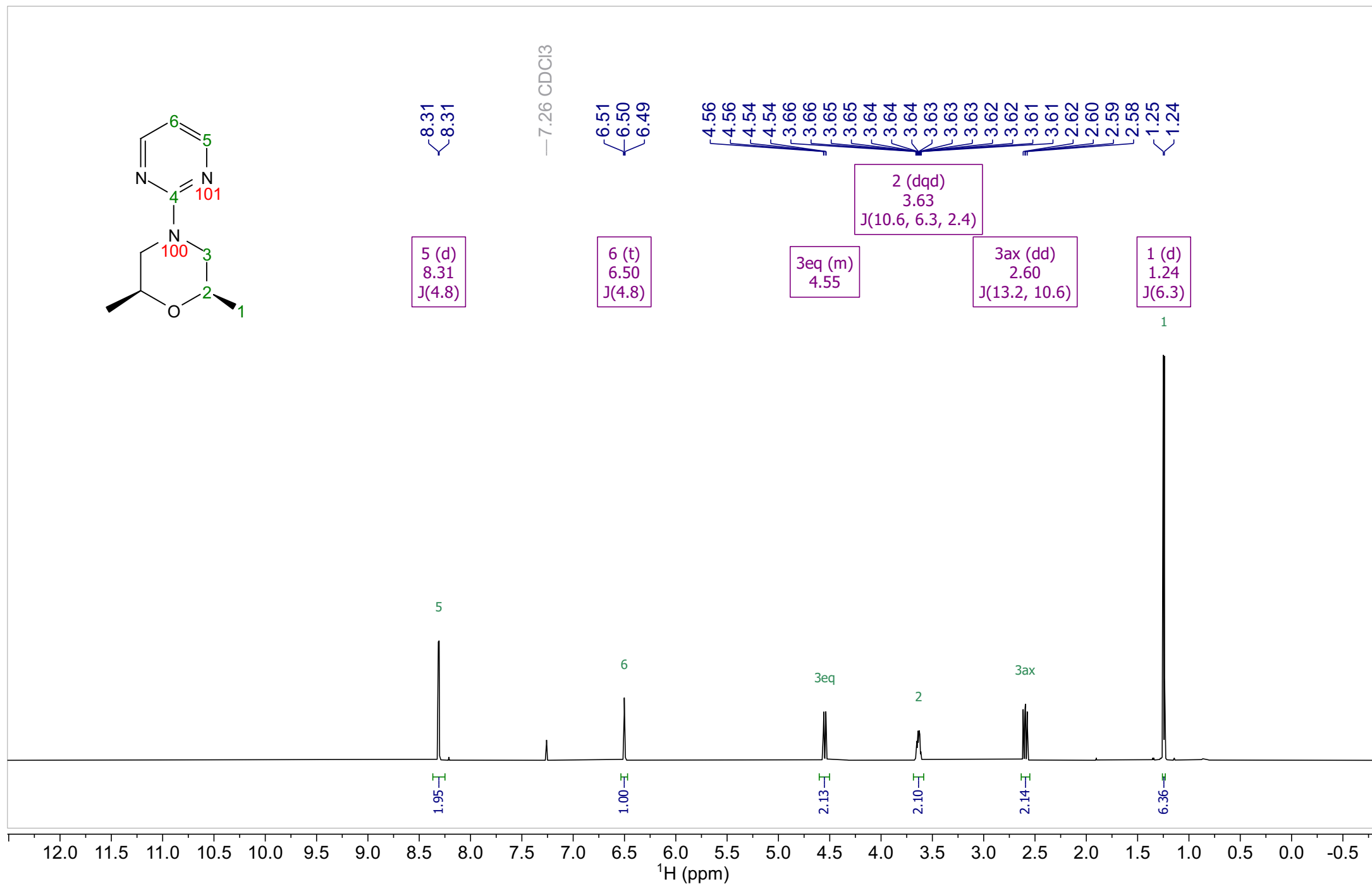

$^{13}\text{C}$  NMR (151 MHz,  $\text{CDCl}_3$ )  $\delta$  161.1, 157.8, 110.1, 71.9, 49.5, 19.0.

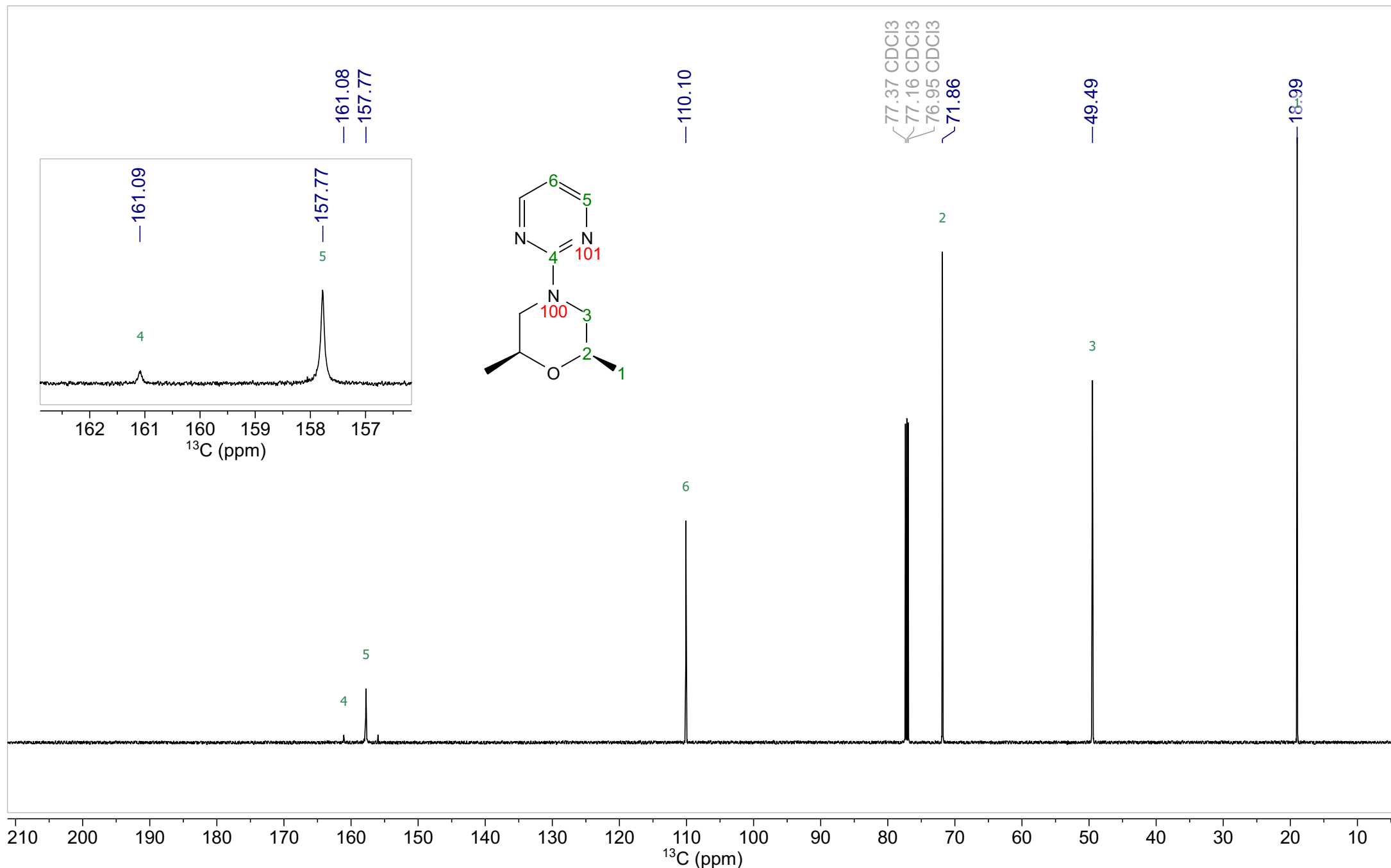

Supplement: Supplementary file 5 [file e-82-00768-sup5.pdf]
