# Supplementary material for: Crystal structure of the meso compound (2R,6S)-4-(5-bromo­pyrimidin-2-yl)-2,6-di­methyl­morpholine
Source: Acta Crystallogr E Crystallogr Commun. 2026 Jun 23;82(Pt 7):768–72. doi: 10.1107/S2056989026006158 (PMC13330847; doi:10.1107/S2056989026006158)
Supplement: Supplementary file 6 [file e-82-00768-sup6.pdf]

^{1}H NMR (402 MHz, DMSO) δ 8.45 (s, 2H, H5), 4.45 – 4.33 (m, 2H, H3eq), 3.53 (dqd, J = 10.7, 6.2, 2.5 Hz, 2H, H2), 2.52 (dd, J = 13.2, 10.7 Hz, 2H, H3ax), 1.13 (d, J = 6.2 Hz, 6H, H1).

2025-09-05 — Palme_2532-34 — PRP252 —

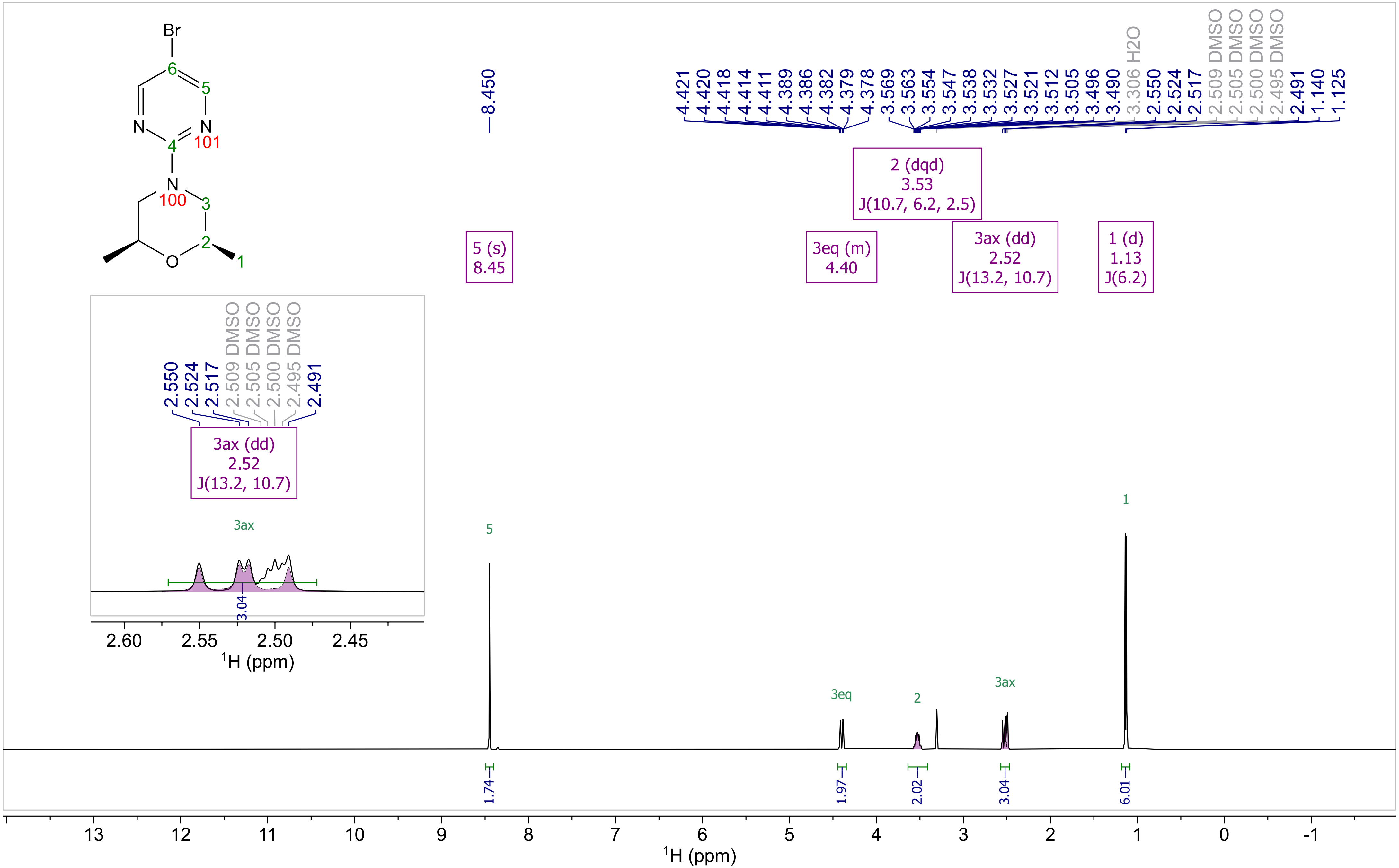

^{13}C-APT NMR (101 MHz, DMSO) δ 159.2 (C4), 157.9 (C5), 105. 5(C6), 70.8 (C2), 48.9 (C3), 18.6 (C1).

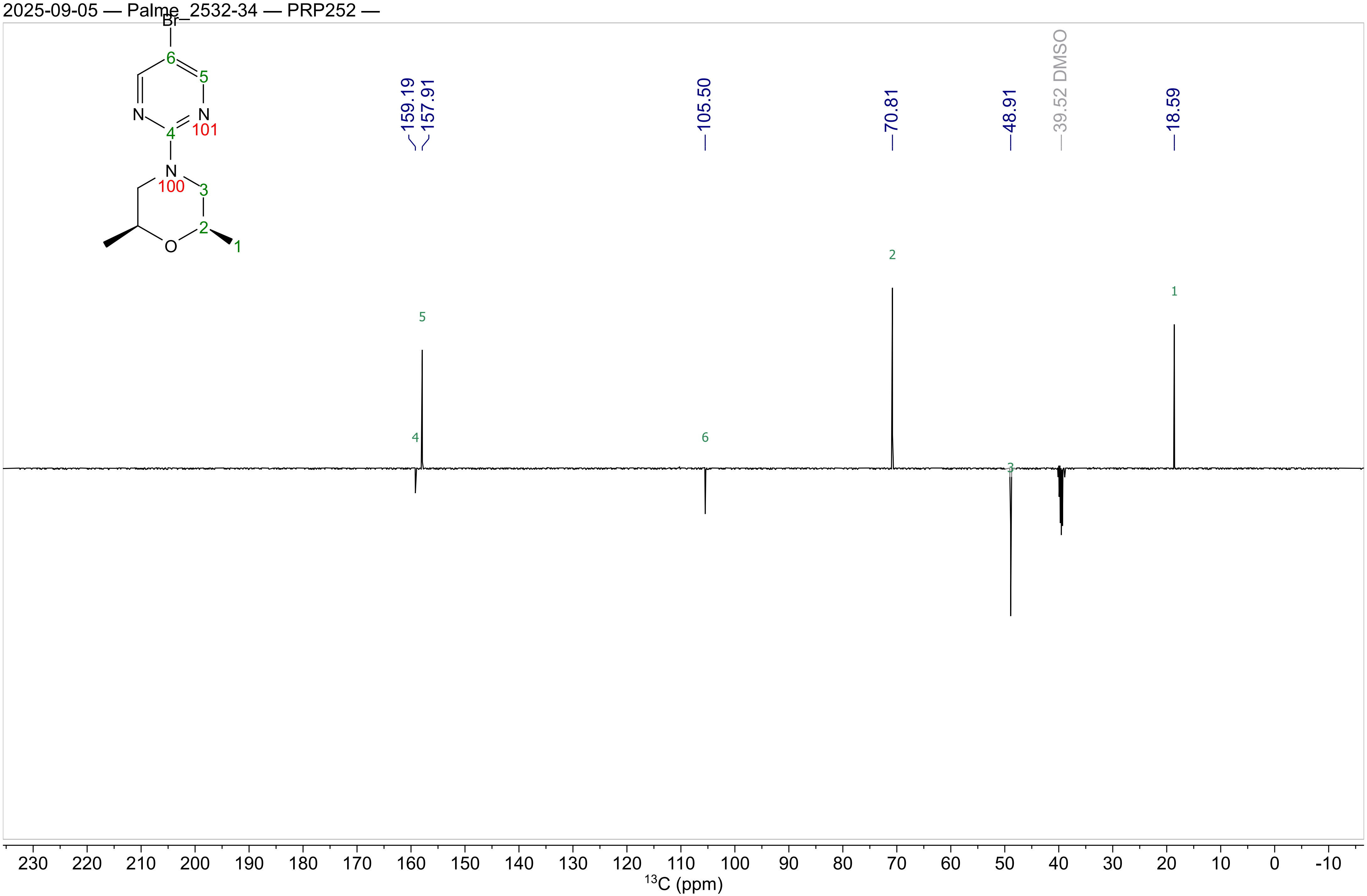

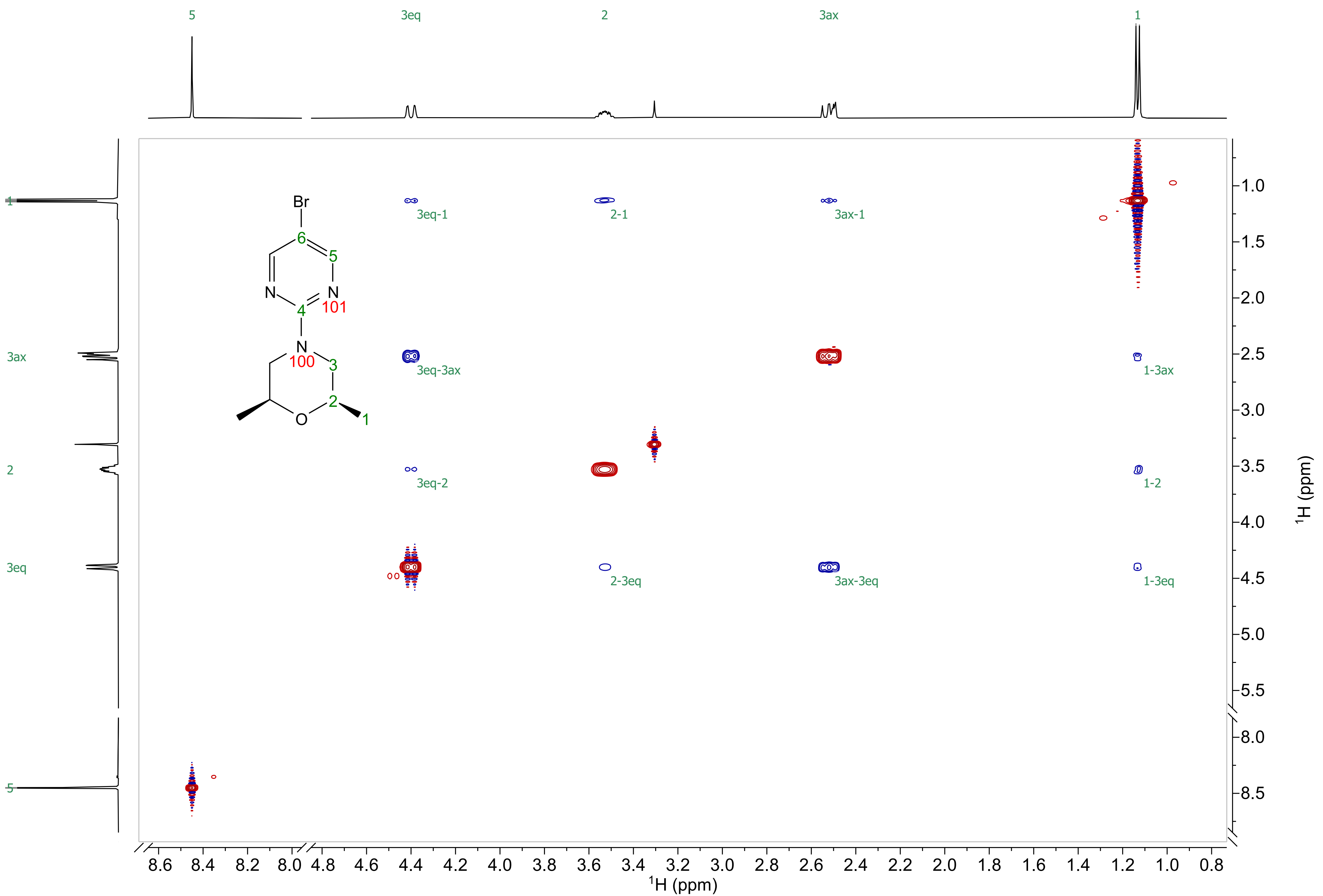

Palme_2532-34_NOESY_2025-09-05_01 — 2025-09-05 — Palme_2532-34 — PRP252 —
